# Automated Opportunistic Trabecular Volumetric Bone Mineral Density Extraction Outperforms Manual Measurements for the Prediction of Vertebral Fractures in Routine CT

**DOI:** 10.3390/diagnostics13122119

**Published:** 2023-06-20

**Authors:** Sophia S. Goller, Jon F. Rischewski, Thomas Liebig, Jens Ricke, Sebastian Siller, Vanessa F. Schmidt, Robert Stahl, Julian Kulozik, Thomas Baum, Jan S. Kirschke, Sarah C. Foreman, Alexandra S. Gersing

**Affiliations:** 1 Department of Radiology, University Hospital, LMU Munich, Marchioninistr. 15, 81377 Munich, Germany; jens.ricke@med.uni-muenchen.de (J.R.); vanessa.schmidt@med.uni-muenchen.de (V.F.S.); 2 Institute for Diagnostic and Interventional Neuroradiology, University Hospital, LMU Munich, Marchioninistr. 15, 81377 Munich, Germany; jon.rischewski@tum.de (J.F.R.); thomas.liebig@med.uni-muenchen.de (T.L.); robert.stahl@med.uni-muenchen.de (R.S.); alexandra.gersing@med.uni-muenchen.de (A.S.G.); 3 Department of Neurosurgery, University Hospital, LMU Munich, Marchioninistr. 15, 81377 Munich, Germany; sebastian.siller@med.uni-muenchen.de; 4 Institute of Micro Technology and Medical Device Technology (MIMED), Technical University of Munich, Boltzmannstr. 15, 85748 Garching, Germany; julian.kulozik@tum.de; 5 Department of Diagnostic and Interventional Neuroradiology, Klinikum Rechts der Isar, Technical University of Munich, Ismaninger Str. 22, 81675 Munich, Germany; thomas.baum@tum.de (T.B.); jan.kirschke@tum.de (J.S.K.); 6 Department of Diagnostic and Interventional Radiology, Klinikum Rechts der Isar, Technical University of Munich, Ismaninger Str. 22, 81675 Munich, Germany; sarah.foreman@tum.de

**Keywords:** osteoporosis, opportunistic screening, computed tomography, bone mineral density, osteoporotic fractures, computational neural networks

## Abstract

Opportunistic osteoporosis screening using multidetector CT-scans (MDCT) and convolutional neural network (CNN)-derived segmentations of the spine to generate volumetric bone mineral density (vBMD) bears the potential to improve incidental osteoporotic vertebral fracture (VF) prediction. However, the performance compared to the established manual opportunistic vBMD measures remains unclear. Hence, we investigated patients with a routine MDCT of the spine who had developed a new osteoporotic incidental VF and frequency matched to patients without incidental VFs as assessed on follow-up MDCT images after 1.5 years. Automated vBMD was generated using CNN-generated segmentation masks and asynchronous calibration. Additionally, manual vBMD was sampled by two radiologists. Automated vBMD measurements in patients with incidental VFs at 1.5-years follow-up (*n* = 53) were significantly lower compared to patients without incidental VFs (*n* = 104) (83.6 ± 29.4 mg/cm^3^ vs. 102.1 ± 27.7 mg/cm^3^, *p* < 0.001). This comparison was not significant for manually assessed vBMD (99.2 ± 37.6 mg/cm^3^ vs. 107.9 ± 33.9 mg/cm^3^, *p* = 0.30). When adjusting for age and sex, both automated and manual vBMD measurements were significantly associated with incidental VFs at 1.5-year follow-up, however, the associations were stronger for automated measurements (β = −0.32; 95% confidence interval (CI): −20.10, 4.35; *p* < 0.001) compared to manual measurements (β = −0.15; 95% CI: −11.16, 5.16; *p* < 0.03). In conclusion, automated opportunistic measurements are feasible and can be useful for bone mineral density assessment in clinical routine.

## 1. Introduction

Osteoporosis is a systemic bone disease characterized by a decrease in bone mass and microarchitectural deterioration of bone tissue, predisposing the individual to an increased risk of osseous fractures [1]. Osteoporosis occurs very frequently worldwide, yet particularly affects the elderly population in developed countries [2]. The prevalence of osteoporotic vertebral fractures (VFs) amounts to between 18% and 26% among Europeans older than 50 years [3]. VFs can lead to severe consequences, such as a reduced quality of life [4], a two-fold increase in age-adjusted mortality risk [5], and a three-fold increase in the risk of developing additional fractures compared to the normal population [6]. Early medical treatment of osteoporotic patients with a high fracture risk is, therefore, highly recommended in order to avoid poor outcomes [7,8]. However, osteoporosis is an underdiagnosed condition in clinical practice. Osteoporotic patients are commonly asymptomatic until a fracture occurs, and even among fractures, only about one quarter of osteoporotic VFs are diagnosed clinically [9,10]. Thus, it is a high priority in the care of osteoporotic patients to identify individuals at high fracture risk in order to timely initiate medical treatment before the first fracture occurs. 

Dual-energy X-ray absorptiometry (DXA) measuring the areal bone mineral density (aBMD) is currently considered the standard screening method for osteoporosis beside assessing clinical risk factors [11]. Yet, there are some major concerns regarding this approach. Only 44% of females and 21% of males with osteoporotic fractures exhibited a low aBMD in a large observational study [12], which further emphasizes the inherent inaccuracies of DXA [13].

The potential of opportunistic vBMD values derived from routine CT data have increasingly moved into the focus of osteoporosis screening [14,15]. Important disadvantages of the established method are a user-dependent and time-consuming extraction process of vBMD [16,17,18]. A deep-learning (DL)-based approach recently enabled the automated extraction of vBMD from multidetector CT (MDCT) scans [19,20,21,22,23] and has demonstrated an improvement of the association between VFs and vBMD compared to the DXA method [20].

Already published studies investigating machine learning and CNN approaches to predict future osteoporotic fractures are mainly risk assessment tools requiring the input of existing clinical examination data, such as aBMD derived from DXA, but not generating any new BMD data for fracture prediction [24,25,26]. Other approaches focused on machine learning combined with texture analysis of vertebrae, however, not did not take BMD into consideration [27], or performed feature extraction by a deep-learning algorithm from lateral spine radiographs by using non-CNN-generated aBMD values from DXA-scans [28].

Therefore, the aim of this study was to compare opportunistic CT-based trabecular vBMD measurements derived using automated convolutional neural network (CNN)-based segmentations with manual vBMD measurements for the prediction of the occurrence of incidental osteoporotic VFs at 1.5-years follow-up.

## 2. Materials and Methods

**Figure 1:**
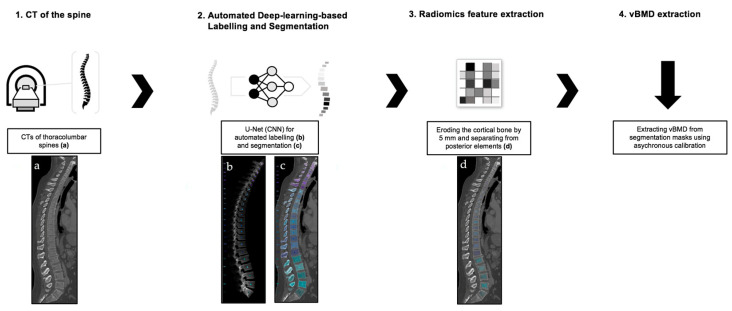
Flowchart illustrating the study’s workflow with regards to automated spine processing and vBMD extraction. Clinical routine MDCT scans of the thoracolumbar spine were retrospectively identified by using criteria for inclusion as described in the Section 2.1 (**1**. **a**). Segmentation and labelling of vertebrae were performed using an CNN-based automated pipeline (https://anduin.bonescreen.de) (**2**. **b**,**c**). Segmentation masks of vertebral bodies were eroded by the cortical bone and posterior vertebral elements were removed using affine and deformable transformations (**3**. **d**). vBMD measurements were extracted using asynchronous calibration and correction for contrast medium, if applicable (**4**).

The workflow utilized in this study is illustrated in Figure 1.

### 2.1. Subjects

The local Ethics Committee of University Hospital, LMU Munich, Marchioninistr. 15, 81,377 Munich, Germany approved this study [protocol code 23-0131, date of approval 03.04.2023] and waived the requirement of written informed consent.

The study population was retrospectively identified within the local picture archiving and communication system (PACS) on all patients registered between August 2009 and April 2021. The criteria for the inclusion of patients with incidental VFs were (1) a history of two MDCT scans showing the thoracolumbar spine (T1 to L5) and (2) at least one new appeared incidental osteoporotic VF in the 1.5-year follow-up CT scan. Each patient with an incidental VF at 1.5-years follow-up was frequency matched to two patients who did not develop an incidental VF between baseline and 1.5-years follow-up. The matching criteria were sex, age (range of 5 years) and time interval between the two required CT scans. Baseline and follow-up scans were routine CTs with other indication than to screen for osteoporosis, of which most were staging CTs in tumor patients. The exclusion criteria were a history of vertebral metastasis or hematologic disorder, traumatic spine injury, a history of an osteoporotic fracture in a different region (e.g., hip), or previous spinal surgery. Incidental osteoporotic VFs were evaluated on the 1.5-year follow-up scans (18.3 ± 14.8 months) using the semi-quantitative technique by Genant, which rates vertebrae from grade 0 to grade 3 depending on the loss of vertebral height [29]. Based on visual image review, patients were categorized as either a patient with newly fractured vertebrae (grade ≥ 1) or without fractured vertebrae.

This yielded a final study cohort of 157 patients (84 females, 73 males) with a mean age of 65.7 ± 11.8 years, with 53 patients (28 women, mean age 64.7 ± 12.0 years) showing at least one incidental VF at the 1.5-year follow-up and 104 patients (56 women) with no evidence of an incidental VF at the 1.5-year follow-up.

### 2.2. CT Image Acquisition

CT data were acquired on six different MDCT scanners (GE Revolution and GE Optima, GE Healthcare; Somatom Definition AS+, Somatom Definition Edge, Somatom Drive, and Somatom Force, Siemens Healthineers). A peak tube voltage of 120 kVp and adaptive tube load was used for all images with the scanners in helical mode. Depending on the clinical indication, most scans were performed after application of intravenous contrast agent (Iomeron 400, Bracco) (*n* = 152). Arterial or portal venous phase scans were either acquired after a CT-attenuation threshold was surpassed in a region of interest (ROI) placed in the aorta (arterial) or after 70 s of delay (venous). Reconstructions for sagittal reformation of the spine were conducted with a standard bone kernel and 2 mm slice thickness. Clinical indications for CT imaging were not related to bone densitometry and included, e.g., acute and chronic back pain, suspected VF, as well as the assessment of acute thoracic and abdominal pathologies.

### 2.3. Opportunistic CT-Based Measurements of Volumetric BMD

Volumetric BMD measures were extracted from clinical baseline MDCT scans in vertebrae L1 to L4 for both automatic and manual analysis in order to assess the average vBMD according to previous study results [20,30,31]. In the case of VFs of L1 to L4 in any CT scans, these vertebral bodies were excluded from data analysis (*n* = 13). No patients with more than one vertebral height from L1 to L4 had to be excluded due to a preexisting VF.

#### 2.3.1. Asynchronous Calibration and Correction for Contrast Medium

CT attenuation in Hounsfield units (HU) was converted to vBMD using asynchronous calibration in a similar manner as previously described by Löffler et al. [20]. In asynchronous calibration, HU–BMD-relations are calculated after scanning phantoms containing bone-equivalent elements with hydroxyl-apatite inserts of known density in milligrams per cubic centimeter (Anthropomorphic Abdomen Phantom, QRM Quality Assurance in Radiology and Medicine). The equations are specific for a certain scanner, respectively acquisition protocol, as previously reported [31]. To correct for BMD bias after the injection of contrast medium, linear correction equations able to correct for arterial and venous contrast phases were applied, analogous to a previously published study [32]. Both manually and automatically sampled vBMD measures were corrected for contrast medium prior to any subsequent evaluation of the data, if applicable.

#### 2.3.2. Automatic and Manual Extraction of Trabecular Volumetric BMD

**Figure 2:**
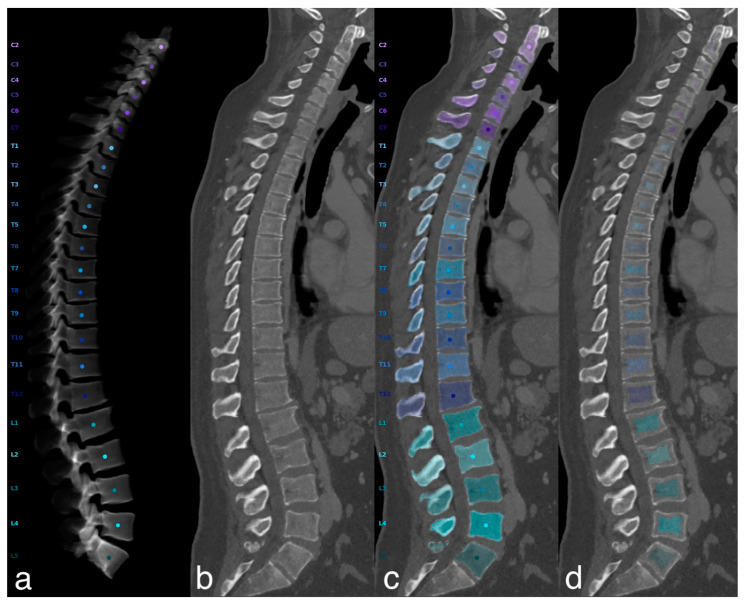
Automatic extraction of trabecular volumetric BMD. Representative example illustrating the automated spine processing and BMD extraction pipeline. CT scan (**b**) of a 42-year-old male visualized as virtual radiograph in lateral projection (**a**) and curved planar reconstructions in lateral views (**c**,**d**). Anduin (https://anduin.bonescreen.de) was used to localize, label, and segment the vertebrae (**c**). To exclude cortical bone, vertebral bodies were separated from the posterior vertebral elements and segmentation masks were eroded by 5 mm (**d**). L1 to L4 yielded a mean trabecular vBMD of 123.7 mg/cm^3^.

**Figure 3:**
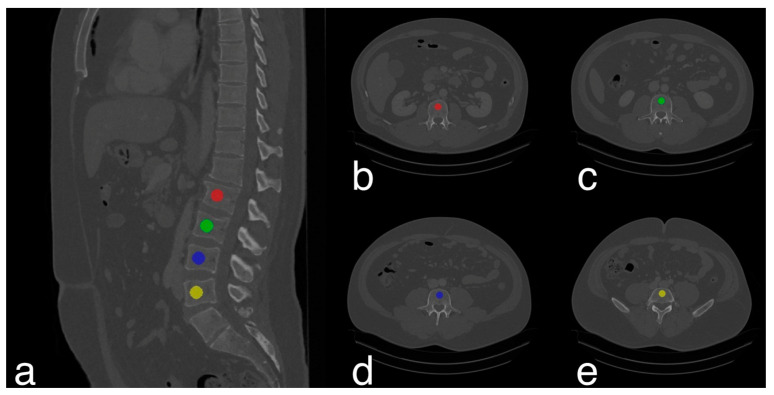
Manual extraction of trabecular volumetric BMD. CT scan of a 42-year-old male illustrating the manual trabecular vBMD extraction process. Volumetric ROIs of 4.5 cm^3^ were manually placed in the anterior trabecular region of vertebrae L1 to L4 (**a**–**e**) yielding a mean trabecular vBMD of 161.3 mg/cm^3^ after converting CT attenuation in HU to BMD values using asynchronous calibration.

An automatic three-step procedure, which was implemented in Python, was used for the extraction of vBMD measures. In a first step, automated segmentation of vertebrae was performed by a CNN-based framework (https://anduin.bonescreen.de) that is able to identify the spine and to create segmentation masks after labelling each vertebral body [20,23,33]. Secondly, as only the vertebral body is used for vBMD extraction, the posterior vertebral elements in the segmentation masks were removed using affine and deformable transformations, for fitting templates of vertebral subregions to each vertebral level. Thirdly, to only sample trabecular vBMD, segmentation masks of vertebral bodies were eroded by the cortical bone [20] (Figure 2). Manual extraction of HUs on the other hand was performed by placing a volumetric ROI of 4.5 cm^3^ in the anterior trabecular region of vertebrae L1 to L4 (Figure 3) and BMD values were calculated from these as described above.

#### 2.3.3. Image Reconstructions for Quality Assurance

For identifying vertebrae that had to be excluded from vBMD assessment, e.g., due to severe degenerative changes leading to alterations in bone mass not associated with osteoporosis, curved planar reconstructions from CT data were generated in sagittal and coronal views. With an opacity of 40%, the reconstructions were overlaid with segmentation masks on the center of mass of the vertebral bodies. In addition, virtual radiographs in the lateral projection were calculated from the CT data (Figure 2).

### 2.4. Clinical Thresholds for Volumetric BMD Measures

For trabecular vBMD assessment, the diagnostic thresholds for osteoporosis (BMD < 80 mg/cm^3^) and osteopenia (80 mg/cm^3^ ≤ BMD ≤ 120 mg/cm^3^) proposed by the American College of Radiology (ACR) were applied [34].

### 2.5. Statistical Analysis

Statistical analysis was performed with SPSS (version 28; IBM SPSS Statistics for macOS, IBM Corp., Armonk, NY, USA) using a two-sided level of significance of 0.05 for all statistical tests. A Shapiro-Wilk test was performed to test for normal distribution of the data. One-way analysis of variance (ANOVA; for parametric testing) and chi-square tests (for categorical variables) were used to evaluate differences in the subject characteristics between patients with and without VFs at follow-up. Due to the large number of parameters, the analyses were categorized into primary and exploratory data. As primary data, based on previous publications [20,30,31,35], multivariable linear regression analyses adjusting for age and sex were performed using the fracture status (occurrence of incidental VF at follow-up versus no incidental VF at follow-up) as a dependent variable and automatic respectively manual vBMD extraction (average across L1–L4) as an independent variables for the assessment of associations between the different vBMD measurement approaches and the incidental VF status. Goodness of fit measures were applied for linear regression analysis including Durbin–Watson statistics. In addition, as exploratory analyses, multivariate linear regression analyses adjusting for age and sex were performed to assess associations between automatic respectively manual vBMD measurements at single vertebral levels from L1 to L4, respectively, at combinations of two consecutive vertebral bodies (L1–L2, L2–L3, and L3–L4) and fracture status at follow-up. Adjustment for age and sex was performed as both parameters have been proven to potentially influence BMD results [36].

## 3. Results

**Figure 4:**
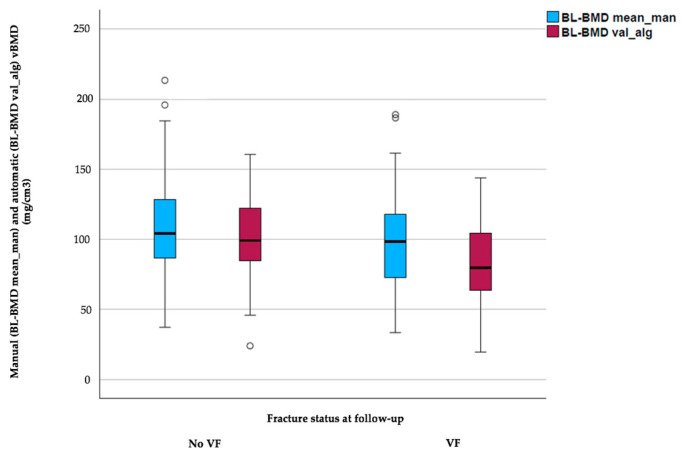
Boxplots showing the minimum, first quartile, median, third quartile, and maximum for both automatic- and manual-derived baseline vBMD values in the patient cohort with and without incidental vertebral fractures at follow-up, respectively.

A total of 157 patients (84 women, 73 men) with a mean age of 65.7 ± 11.8 years were included in this study. Patients with newly occurring incidental VFs at 1.5-year follow-up showed no statistically significant differences in sex distribution and age (64.8 ± 12.0 years vs. 66.3 ± 11.8 years, *p* = 0.77) when compared to controls without incidental VFs at 1.5-year follow-up. Patients with incidental VFs at follow-up (*n* = 53) showed significantly lower automatically extracted average vBMD values across L1 to L4 at baseline (83.6 ± 29.4 mg/cm^3^ vs. 102.1 ± 27.7 mg/cm^3^, *p* < 0.001) compared to patients without incidental VFs at follow-up. Manual average vBMD values assessed at baseline revealed no significant differences between patients with incidental fractures at follow-up (99.2 ± 37.6 mg/cm^3^ vs. 107.9 ± 33.9 mg/cm^3^, *p* = 0.30) compared to the patient cohort without incidental VFs at follow up (Table 1 and Figure 4).

The incidental VF status at follow-up, meaning the occurrence of a new incidental VF between baseline and follow-up scan, was significantly associated with automatically extracted vBMD adjusting for age and sex (β = −0.32; 95% confidence interval (CI): −20.10, 4.35; *p* < 0.001). A significant but less strong association was also found between the manual vBMD assessment and the incidental VF status at follow-up (β = −0.15; 95% CI: −11.16, 5.16; *p* < 0.03).

Further, the incidental VF status at follow-up was significantly associated with automatically extracted vBMD assessment adjusting for age and sex for both single vertebral levels from L1 to L4 (L1: *p* < 0.001, L2: *p* < 0.001, L3: *p* = 0.001, L4: *p* = 0.002) as well as for combinations of consecutive vertebral body heights from L1-L2 to L3-L4 (L1-2: *p* < 0.001, L2-3: *p* < 0.001, L3-4: *p* < 0.001) (Table 2 and Table 3). The incidental VF status at follow-up was significantly associated with manual trabecular vBMD assessment adjusting for age and sex at single vertebral levels L1 and L2 (L1: *p* = 0.006, L2: *p* = 0.029) and at consecutive levels L1-L2 and L2-L3 (L1-2: *p* < 0.001, L2-3: *p* = 0.007), respectively, while there was neither a significant association found at single vertebral levels L3 and L4 nor at the combination of the BMD values from L3 to L4 (Table 2 and Table 3).

## 4. Discussion

Osteoporosis is a highly prevalent disorder in the elderly characterized by decreased BMD and microarchitectural deterioration of the bone [1,37]. This leads to an increased risk of low-energy trauma fractures, of which vertebral fractures are the most common [3]. Incidental osteoporotic fractures cause a significant decrease in quality of life [4], increased risk of future fractures [6], and bring burden to the socioeconomic system [38]. As an underdiagnosed condition [9,10], diagnostic tools for an early discovery of patients at risk for an incidental osteoporotic fracture are important. The gold standard tool for the diagnosis of osteoporosis is DXA, measuring aBMD [11]. This highlights a major problem of DXA, measuring areal but not volumetric BMD, which is easily contaminated by intra- and extra-osseous soft tissue effects [13]. Avoiding this, qCT is a different, more accurate method for osteoporosis assessment, measuring vBMD from dedicated CT-scans performed to assess BMD, but has the disadvantage of additional radiation and costs [39]. Therefore, we focused on using opportunistically generated vBMD by a CNN-based approach, causing no additional radiation to the patient and minimizing the costs for future vertebral fracture prediction.

Automated and manual opportunistic trabecular vBMD extractions of the lumbar vertebrae L1 to L4 were performed in clinical routine MDCT scans to assess the association between the vBMD and the development of incidental VFs of the thoracolumbar spine. The occurrence of new incidental VFs at 1.5-years follow-up was significantly associated with automated vBMD measurements averaged across L1 to L4, whereas there was a less significant association found between the average manual vBMD (L1-L4) and incidental VF status at 1.5-years follow-up. Patients with new incidental VFs showed significantly lower automatically assessed vBMD values in the baseline CT scan compared to patients without incidental VFs, yet this comparison did not reach the level of significance when assessing the manually extracted BMD. Further, the incidental VF status at the 1.5-year follow-up was significantly associated with automatically extracted vBMD for both single vertebral levels from L1 to L4 as well as for combinations of consecutive vertebral bodies from L1–L2 to L3–L4, yet the manually assessed vBMD values at the single vertebral levels L3 and L4 and the combination of L3 to L4 showed no significant associations.

Unlike DXA, CT enables the extraction of vBMD since it is a three-dimensional imaging modality. The ACR defined reference values to allow the use of CT data for standardized diagnosis of osteopenia (80 ≤ BMD ≤ 120 mg/cm^3^) and osteoporosis (BMD < 80 mg/cm^3^) for the lumbar spine [34] relating to measurements within trabecular bone tissue [34,40]. By now, quantitative computed tomography (QCT) is the imaging modality used to calculate the vBMD with dedicated software packages typically derived from three consecutive vertebral levels (L1 to L3) [14,41]. This is commonly achieved by measuring a reference phantom with known density during the same scanning process to be able to convert attenuation values in HU to vBMD [14,41]. A fundamentally different method for vBMD assessment is using CT data acquired for other clinical indications than osteoporosis screening, e.g., such as oncological staging, which is referred to as opportunistic CT [14,41]. A major benefit with this approach is that additional scanning time respective to radiation exposure can be saved [14,41]. In particular, patients who regularly undergo CT examinations, such as cancer patients, and have an increased risk of osteoporosis due to either comorbidity or treatment side effects may mostly benefit from opportunistic osteoporosis screening [42].

Various study results have led to an increasing acceptance of opportunistic osteoporosis assessment using clinical routine CT data over recent years [15,16,17,20,22,30,43,44,45,46,47,48,49]. QCT and opportunistically used CT data showed no significant difference in vBMD measurements, underlining the fact that opportunistic bone densitometry is feasible for both non-contrast as well as contrast-enhanced CT-scans [46]. Opportunistic trabecular vBMD can either be manually determined by placing small volumetric ROIs in vertebral bodies, which is relatively time consuming and more user-dependent [18] than an automated pipeline, which enables vertebral segmentation, asynchronous calibration for HU-to-vBMD conversion, and correction of contrast medium within several seconds [30]. The automated technique with vertebral segmentation of the entire trabecular region sparing the cortical bone (Figure 3) covers significantly more anatomically involved structure than the established manual method [50,51]. This could explain the less strong association between incidental VF status at the 1.5-year follow-up and manually assessed vBMD averaged across L1 to L4 compared to incidental VF status and the vBMD averaged across L1 to L4 derived from automatic measurements. Another explanation could be the higher user-dependency in manually extracting vBMD compared to the automatic CNN approach [16].

A previous study revealed significantly lower trabecular vBMD values in patients with VFs than in controls without VF analyzing the diagnostic accuracy of vBMD threshold values at different spinal levels derived from opportunistic routine CT data for the prediction of incident VFs of the thoracolumbar spine, which is in line with the results presented in this study [30]. In addition, multiple studies revealed comparable results of lower vBMD values in patients with VFs for opportunistically used CT, with varying but overall acceptable discriminatory power to differentiate patients with and without VFs in different study settings [43,44,45]. Automated opportunistic osteoporosis screening of vBMD along the thoracolumbar spine allowing for risk assessment of imminent VFs and level-specific thresholds at the thoracolumbar spine for the identification of patients at high fracture risk have recently been introduced [30]. In addition, an important previous study comparing different spinal bone measures derived from automatic as well as manual assessment in routine CT and DXA for evaluating in their association with prevalent osteoporotic VFs using the same fully automated pipeline showed that except for bone mineral content, all CT-based measures performed significantly better as predictors for VFs compared to DXA [20]. In some ways different from our findings, age- and sex-adjusted associations with fracture status were strongest for manually assessed trabecular vBMD followed by automatically assessed vBMD [20]. However, the design, objectives, and methods of this study are not fully comparable with ours as the authors assessed the association of spinal bone measures with prevalent osteoporotic VFs, while we analyzed incidental VFs. Evaluating the prediction of incident VFs using CT-based finite element analysis, Allaire et al. showed an association between vertebral strength measures and incident vertebral fractures, which is in accordance with our findings [52]. However, the patient cohort of the cited study was significantly lower in the previous study [52]. A further previous study analyzed incidental VFs of elderly men and reported stronger associations with fracture risk for trabecular vBMD than cortical vBMD, which may be due to a greater metabolic activity of the trabecular compartment [53]. This is in line with our study, in which trabecular vBMD was assessed and showed an association with the presence of incidental VFs at 1.5-year follow-up in a longitudinal analysis, when comparing patients matched for age and sex with and without incidental VFs.

In a future perspective, automated opportunistic vBMD extraction could be added to more routine MDCT scans in groups with increased fracture risk as well as pediatric cohorts who receive regular MDCT-scans, to timely initiate medical or surgical treatment if needed. Especially due to the correlation of low bone quality in early childhood and the increased risk for the development and progression of scoliosis [54], early automated opportunistic detection of low vBMD could be helpful to diagnose and, therefore, treat such conditions earlier. This is may potentially prevent future fractures and has to be further investigated.

We acknowledge that this study has limitations. We used a retrospective study design. Therefore, we could not correlate laboratory parameters such as vitamin D or serum calcium levels with the vBMD measures due to lacking patient blood samples, even though low bone mass quantified by vitamin D and calcium showed an increased low-energy trauma fracture risk in a pediatric population [55]. Thus, future research could address the correlation of automated vBMD measures with serum vitamin D and calcium levels in an adult study cohort. As not every patient of the study cohort additionally underwent DXA or QCT at the time of baseline MDCT, relevant radiological parameters, such as phantom-calibrated vBMD or aBMD values could not be assessed. Baseline and follow-up CT scans were partially performed at different scanners with slight differences in image protocols and software. However, this exactly reflects the clinical routine setting.

## 5. Conclusions

This study shows a significant association between automated CNN-based measurements of opportunistic trabecular vBMD and incidental VFs at the thoracolumbar spine at 1.5-years follow-up, with potential superiority to manual vBMD measures. Therefore, automated vBMD measurements could be highly advantageous in clinical practice for identifying individuals at a high risk of an incidental vertebral fracture, without additional costs and radiation exposure.

## Figures and Tables

**Table 1:** Baseline characteristics of patients stratified by vertebral fracture status; VF, vertebral fracture (at follow-up); vBMD, volumetric bone mineral density; SD, standard deviation; Significant results (***p* < 0.05**) are bolded.

Variable	VF (*n* = 53)	No VF (*n* = 104)	VF vs. No VF *p*-Value	Total (*n* = 157)
Females, n (%)	28 (53%)	56 (54%)	0.63	84 (54%)
Age, years, mean (SD)	64.7 (12.0)	66.3 (11.7)	0.77	65.7 (11.8)
Automatic vBMD *, mg/cm^3^, mean (SD)	83.6 (29.4)	102.1 (27.7)	<0.001	95.9 (29.5)
Manual vBMD *, mg/cm^3^, mean (SD)	99.2 (37.6)	107.9 (33.9)	0.30	105.0 (35.3)

* trabecular vBMD.

**Table 2:** Single vertebra analysis. Associations between automatically and manually assessed trabecular vBMD and incident vertebral fracture status at follow-up for all single vertebral levels of the lumbar spine (L1 to L4); Multivariable linear regression adjusting for age and sex.

Level	Automatic vBMD	*p*-Value	Manual vBMD	*p*-Value
L1	−0.33 (−0.34–−0.32)	**<0.001**	−0.22 (−0.23–−0.21)	**0.006**
L2	−0.30 (−0.31–−0.29)	**<0.001**	−0.18 (−0.19–−0.17)	**0.029**
L3	−0.25 (−0.26–−0.24)	**0.001**	−0.13 (−0.14–−0.12)	0.11
L4	−0.24 (−0.25–−0.23)	**0.002**	−0.07 (−0.08–−0.06)	0.38

Numbers are given as standardized regression coefficients (β) and 95% confidence intervals (95%-CI); vBMD, volumetric bone mineral density; significant results (***p* < 0.05**) are bolded. Goodness of fit measures were applied for both linear regression models (manual: R^2^ = 0.27, *p* < 0.001; Durbin–Watson statistic 2.06; automatic: R^2^ = 0.25, *p* < 0.001; Durbin–Watson statistic 2.07).

**Table 3:** Analysis of averages across combinations of two consecutive vertebral bodies: Associations between automatically and manually assessed trabecular vBMD and incident vertebral fracture status at follow-up for combinations of consecutive vertebral body levels of the lumbar spine (L1–L2 to L3–L4); Multivariable linear regression adjusting for age and sex.

Level	Automatic vBMD	*p*-Value	Manual vBMD	*p*-Value
L1–L2	−0.32 (−0.33–−0.31)	**<0.001**	−0.20 (−0.21–−0.19)	**<0.001**
L2–L3	−0.28 (−0.29–−0.27)	**<0.001**	−0.15 (−0.16–−0.14)	**0.007**
L3–L4	−0.25 (−0.26–−0.24)	**<0.001**	−0.10 (−0.11–−0.09)	0.08

Numbers are given as standardized regression coefficients (β) and 95% confidence intervals (95%-CI); vBMD, volumetric bone mineral density; significant results (***p* < 0.05**) are bolded. Goodness of fit measures were applied for both linear regression models (manual: R^2^ = 0.27, *p* < 0.001; Durbin–Watson statistic 2.06; automatic: R^2^ = 0.25, *p* < 0.001; Durbin–Watson statistic 2.07).

## Data Availability

Data supporting the present study are available from the corresponding author upon reasonable request.

## References

[1] NIH Consensus Development Panel on Osteoporosis Prevention, Diagnosis, and Therapy (2001). Osteoporosis Prevention, Diagnosis, and Therapy. JAMA.

[2] Johnell O., Kanis J.A. (2006). An estimate of the worldwide prevalence and disability associated with osteoporotic fractures. Osteoporos. Int..

[3] Ballane G., Cauley J.A., Luckey M.M., El-Hajj Fuleihan G. (2017). Worldwide prevalence and incidence of osteoporotic vertebral fractures. Osteoporos. Int..

[4] Hallberg I., Bachrach-Lindstrom M., Hammerby S., Toss G., Ek A.C. (2009). Health-related quality of life after vertebral or hip fracture: A seven-year follow-up study. BMC Musculoskelet. Disord..

[5] Bliuc D., Nguyen N.D., Milch V.E., Nguyen T.V., Eisman J.A., Center J.R. (2009). Mortality risk associated with low-trauma osteoporotic fracture and subsequent fracture in men and women. JAMA.

[6] Melton L.J., Atkinson E.J., Cooper C., O’Fallon W.M., Riggs B.L. (1999). Vertebral fractures predict subsequent fractures. Osteoporos. Int..

[7] Center J.R. (2017). Fracture Burden: What Two and a Half Decades of Dubbo Osteoporosis Epidemiology Study Data Reveal About Clinical Outcomes of Osteoporosis. Curr. Osteoporos. Rep..

[8] Compston J.E., McClung M.R., Leslie W.D. (2019). Osteoporosis. Lancet.

[9] Chesnut C.H. (2001). Osteoporosis, an underdiagnosed disease. JAMA.

[10] Fink H.A., Milavetz D.L., Palermo L., Nevitt M.C., Cauley J.A., Genant H.K., Black D.M., Ensrud K.E., Fracture Intervention Trial Research G. (2005). What proportion of incident radiographic vertebral deformities is clinically diagnosed and vice versa?. J. Bone Miner. Res..

[11] (1993). Consensus development conference: Diagnosis, prophylaxis, and treatment of osteoporosis. Am. J. Med..

[12] Schuit S.C., van der Klift M., Weel A.E., de Laet C.E., Burger H., Seeman E., Hofman A., Uitterlinden A.G., van Leeuwen J.P., Pols H.A. (2004). Fracture incidence and association with bone mineral density in elderly men and women: The Rotterdam Study. Bone.

[13] Bolotin H.H. (2007). DXA in vivo BMD methodology: An erroneous and misleading research and clinical gauge of bone mineral status, bone fragility, and bone remodelling. Bone.

[14] Löffler M.T., Sollmann N., Mei K., Valentinitsch A., Noël P.B., Kirschke J.S., Baum T. (2020). X-ray-based quantitative osteoporosis imaging at the spine. Osteoporos. Int..

[15] Engelke K., Chaudry O., Bartenschlager S. (2023). Opportunistic Screening Techniques for Analysis of CT Scans. Curr. Osteoporos. Rep..

[16] Sollmann N., Loffler M.T., El Husseini M., Sekuboyina A., Dieckmeyer M., Ruhling S., Zimmer C., Menze B., Joseph G.B., Baum T. (2022). Automated Opportunistic Osteoporosis Screening in Routine Computed Tomography of the Spine: Comparison With Dedicated Quantitative CT. J. Bone Miner. Res..

[17] Pickhardt P.J., Lee L.J., del Rio A.M., Lauder T., Bruce R.J., Summers R.M., Pooler B.D., Binkley N. (2011). Simultaneous Screening for Osteoporosis at CT Colonography: Bone Mineral Density Assessment Using MDCT Attenuation Techniques Compared With the DXA Reference Standard. J. Bone Miner. Res..

[18] Pickhardt P.J., Pooler B.D., Lauder T., del Rio A.M., Bruce R.J., Binkley N. (2013). Opportunistic Screening for Osteoporosis Using Abdominal Computed Tomography Scans Obtained for Other Indications. Ann. Intern. Med..

[19] Yasaka K., Akai H., Kunimatsu A., Kiryu S., Abe O. (2020). Prediction of bone mineral density from computed tomography: Application of deep learning with a convolutional neural network. Eur. Radiol..

[20] Loffler M.T., Jacob A., Scharr A., Sollmann N., Burian E., El Husseini M., Sekuboyina A., Tetteh G., Zimmer C., Gempt J. (2021). Automatic opportunistic osteoporosis screening in routine CT: Improved prediction of patients with prevalent vertebral fractures compared to DXA. Eur. Radiol..

[21] Loffler M.T., Sollmann N., Burian E., Bayat A., Aftahy K., Baum T., Meyer B., Ryang Y.M., Kirschke J.S. (2020). Opportunistic Osteoporosis Screening Reveals Low Bone Density in Patients With Screw Loosening After Lumbar Semi-Rigid Instrumentation: A Case-Control Study. Front. Endocrinol..

[22] Ruhling S., Scharr A., Sollmann N., Wostrack M., Loffler M.T., Menze B., Sekuboyina A., El Husseini M., Braren R., Zimmer C. (2022). Proposed diagnostic volumetric bone mineral density thresholds for osteoporosis and osteopenia at the cervicothoracic spine in correlation to the lumbar spine. Eur. Radiol..

[23] Sekuboyina A., Husseini M.E., Bayat A., Loffler M., Liebl H., Li H., Tetteh G., Kukacka J., Payer C., Stern D. (2021). VerSe: A Vertebrae labelling and segmentation benchmark for multi-detector CT images. Med. Image Anal..

[24] Wang Y., Zhang Z., Cai N., Zhou Y., Xiao D. (2018). A Prediction Model for the Risk of Osteoporosis Fracture in the Elderly Based on a Neural Network.

[25] de Vries B.C.S., Hegeman J.H., Nijmeijer W., Geerdink J., Seifert C., Groothuis-Oudshoorn C.G.M. (2021). Comparing three machine learning approaches to design a risk assessment tool for future fractures: Predicting a subsequent major osteoporotic fracture in fracture patients with osteopenia and osteoporosis. Osteoporos. Int..

[26] Kong S.H., Ahn D., Kim B.R., Srinivasan K., Ram S., Kim H., Hong A.R., Kim J.H., Cho N.H., Shin C.S. (2020). A Novel Fracture Prediction Model Using Machine Learning in a Community-Based Cohort. JBMR Plus.

[27] Muehlematter U.J., Mannil M., Becker A.S., Vokinger K.N., Finkenstaedt T., Osterhoff G., Fischer M.A., Guggenberger R. (2019). Vertebral body insufficiency fractures: Detection of vertebrae at risk on standard CT images using texture analysis and machine learning. Eur. Radiol..

[28] Kong S.H., Lee J.W., Bae B.U., Sung J.K., Jung K.H., Kim J.H., Shin C.S. (2022). Development of a Spine X-Ray-Based Fracture Prediction Model Using a Deep Learning Algorithm. Endocrinol. Metab..

[29] Genant H.K., Wu C.Y., van Kuijk C., Nevitt M.C. (1993). Vertebral fracture assessment using a semiquantitative technique. J. Bone Miner. Res..

[30] Dieckmeyer M., Loffler M.T., El Husseini M., Sekuboyina A., Menze B., Sollmann N., Wostrack M., Zimmer C., Baum T., Kirschke J.S. (2022). Level-Specific Volumetric BMD Threshold Values for the Prediction of Incident Vertebral Fractures Using Opportunistic QCT: A Case-Control Study. Front. Endocrinol..

[31] Loffler M.T., Jacob A., Valentinitsch A., Rienmuller A., Zimmer C., Ryang Y.M., Baum T., Kirschke J.S. (2019). Improved prediction of incident vertebral fractures using opportunistic QCT compared to DXA. Eur. Radiol..

[32] Kaesmacher J., Liebl H., Baum T., Kirschke J.S. (2017). Bone Mineral Density Estimations From Routine Multidetector Computed Tomography: A Comparative Study of Contrast and Calibration Effects. J. Comput. Assist. Tomogr..

[33] Loffler M.T., Sekuboyina A., Jacob A., Grau A.L., Scharr A., El Husseini M., Kallweit M., Zimmer C., Baum T., Kirschke J.S. (2020). A Vertebral Segmentation Dataset with Fracture Grading. Radiol. Artif. Intell..

[34] American College of Radiology ACR-SPR-SSR Practice Parameter for the Performance of Muskuloskeletal Quantitative Computed Tomography (QCT). https://www.acr.org/-/media/ACR/Files/Practice-Parameters/QCT.pdf?la.

[35] Dieckmeyer M., Sollmann N., El Husseini M., Sekuboyina A., Loffler M.T., Zimmer C., Kirschke J.S., Subburaj K., Baum T. (2021). Gender-, Age- and Region-Specific Characterization of Vertebral Bone Microstructure Through Automated Segmentation and 3D Texture Analysis of Routine Abdominal CT. Front. Endocrinol..

[36] Havill L.M., Mahaney M.C., T L.B., Specker B.L. (2007). Effects of genes, sex, age, and activity on BMC, bone size, and areal and volumetric BMD. J. Bone Miner. Res..

[37] Lupsa B.C., Insogna K. (2015). Bone Health and Osteoporosis. Endocrinol. Metab. Clin. N. Am..

[38] Johnell O. (1997). The socioeconomic burden of fractures: Today and in the 21st century. Am. J. Med..

[39] Lochmuller E.M., Burklein D., Kuhn V., Glaser C., Muller R., Gluer C.C., Eckstein F. (2002). Mechanical strength of the thoracolumbar spine in the elderly: Prediction from in situ dual-energy X-ray absorptiometry, quantitative computed tomography (QCT), upper and lower limb peripheral QCT, and quantitative ultrasound. Bone.

[40] Oftadeh R., Perez-Viloria M., Villa-Camacho J.C., Vaziri A., Nazarian A. (2015). Biomechanics and mechanobiology of trabecular bone: A review. J. Biomech. Eng..

[41] Link T.M., Kazakia G. (2020). Update on Imaging-Based Measurement of Bone Mineral Density and Quality. Curr. Rheumatol. Rep..

[42] Pfeilschifter J., Diel I.J. (2000). Osteoporosis due to cancer treatment: Pathogenesis and management. J. Clin. Oncol..

[43] Bauer J.S., Henning T.D., Mueller D., Lu Y., Majumdar S., Link T.M. (2007). Volumetric quantitative CT of the spine and hip derived from contrast-enhanced MDCT: Conversion factors. Am. J. Roentgenol..

[44] Baum T., Muller D., Dobritz M., Rummeny E.J., Link T.M., Bauer J.S. (2011). BMD measurements of the spine derived from sagittal reformations of contrast-enhanced MDCT without dedicated software. Eur. J. Radiol..

[45] Baum T., Muller D., Dobritz M., Wolf P., Rummeny E.J., Link T.M., Bauer J.S. (2012). Converted lumbar BMD values derived from sagittal reformations of contrast-enhanced MDCT predict incidental osteoporotic vertebral fractures. Calcif. Tissue Int..

[46] Hopper K.D., Wang M.P., Kunselman A.R. (2000). The use of clinical CT for baseline bone density assessment. J. Comput. Assist. Tomogr..

[47] Link T.M., Koppers B.B., Licht T., Bauer J., Lu Y., Rummeny E.J. (2004). In vitro and in vivo spiral CT to determine bone mineral density: Initial experience in patients at risk for osteoporosis. Radiology.

[48] Burian E., Grundl L., Greve T., Junker D., Sollmann N., Loffler M., Makowski M.R., Zimmer C., Kirschke J.S., Baum T. (2021). Local Bone Mineral Density, Subcutaneous and Visceral Adipose Tissue Measurements in Routine Multi Detector Computed Tomography-Which Parameter Predicts Incident Vertebral Fractures Best?. Diagnostics.

[49] Yeung L.Y., Rayudu N.M., Loffler M., Sekuboyina A., Burian E., Sollmann N., Dieckmeyer M., Greve T., Kirschke J.S., Subburaj K. (2021). Prediction of Incidental Osteoporotic Fractures at Vertebral-Specific Level Using 3D Non-Linear Finite Element Parameters Derived from Routine Abdominal MDCT. Diagnostics.

[50] Roski F., Hammel J., Mei K., Haller B., Baum T., Kirschke J.S., Pfeiffer D., Woertler K., Pfeiffer F., Noel P.B. (2021). Opportunistic osteoporosis screening: Contrast-enhanced dual-layer spectral CT provides accurate measurements of vertebral bone mineral density. Eur. Radiol..

[51] Schwaiger B.J., Gersing A.S., Baum T., Noel P.B., Zimmer C., Bauer J.S. (2014). Bone mineral density values derived from routine lumbar spine multidetector row CT predict osteoporotic vertebral fractures and screw loosening. Am. J. Neuroradiol..

[52] Allaire B.T., Lu D., Johannesdottir F., Kopperdahl D., Keaveny T.M., Jarraya M., Guermazi A., Bredella M.A., Samelson E.J., Kiel D.P. (2019). Prediction of incident vertebral fracture using CT-based finite element analysis. Osteoporos. Int..

[53] Chalhoub D., Orwoll E.S., Cawthon P.M., Ensrud K.E., Boudreau R., Greenspan S., Newman A.B., Zmuda J., Bauer D., Cummings S. (2016). Areal and volumetric bone mineral density and risk of multiple types of fracture in older men. Bone.

[54] Herdea A., Dragomirescu M.C., Ulici A., Lungu C.N., Charkaoui A. (2022). Controlling the Progression of Curvature in Children and Adolescent Idiopathic Scoliosis Following the Administration of Melatonin, Calcium, and Vitamin D. Children.

[55] Herdea A., Ionescu A., Dragomirescu M.C., Ulici A. (2023). Vitamin D-A Risk Factor for Bone Fractures in Children: A Population-Based Prospective Case-Control Randomized Cross-Sectional Study. Int. J. Environ. Res. Public Health.

